# Person-centred ostomy care: a qualitative study of patients’ experiences with routine use of a clinical feedback system during consultations: Word count

**DOI:** 10.1186/s41687-025-00900-6

**Published:** 2025-05-31

**Authors:** Lill Anette Juvik, John Roger Andersen, Kirsten Lerum Indrebø, Anne Marie Sandvoll

**Affiliations:** 1 Department of Surgery, Førde Hospital Trust, Førde, Norway; 2 Department of Health and Caring Sciences, Western Norway University of Applied Sciences, Førde, Norway; 3 Department of Research and Innovation, Førde Hospital Trust, Førde, Norway

**Keywords:** Ostomy care, Routine outcome monitoring, Clinical feedback system, Qualitative research

## Abstract

**Background:**

Adapting to life with an ostomy can be challenging due to significant bodily changes. To better meet patients’ needs and support their adjustment, a clinical feedback system (CFS) with patient-reported outcomes was developed for routine outpatient follow-up consultations with stoma care nurses (SCNs) in specialist health care services. While results from its use prior to consultations are promising, little is known about patients’ perceptions of CFS use in consultations with SCNs. Thus, we aimed to explore how patients experience the routine use of the CFS during follow-up consultations in ostomy care.

**Methods:**

An inductive qualitative design was employed, involving semi-structured individual interviews with 27 patients using the CFS as part of routine care. Data were analysed using reflexive thematic analysis.

**Results:**

The overarching theme, “A flexible, engaged, person-centred follow-up,” was developed, along with four themes: (1) Explicit and implicit use of information in consultations, (2) A springboard for deeper dialogue on sensitive issues, (3) Reassurance that changes in health status will be captured and adequately assessed, and (4) Utility depends on continuity of use. There were variations in how patients experienced their responses being utilised by the SCNs during consultations. Responses were referenced implicitly and explicitly, with a preference for direct communication. Patients found personal value in using the CFS, as it facilitated communication, particularly on sensitive topics. SCNs’ use of the CFS and expertise provided a sense of reassurance when health status was assessed. Regular use of the CFS and a clear understanding of its purpose enhanced its utility and enabled patients to take a more active role in their treatment.

**Conclusion:**

The use of the CFS in ostomy care appears promising. It can improve SCNs’ ability to tailor care to patients’ needs. However, the tool should be applied consistently to realise its full potential in clinical practice.

**Supplementary Information:**

The online version contains supplementary material available at 10.1186/s41687-025-00900-6.

## Introduction

Annually, around 3,700 people in Norway undergo ostomy surgery due to conditions such as inflammatory bowel disease, infections, incontinence and cancer [1, 2]. An ostomy diverts urine or faeces to an external pouch on the abdomen, requiring significant adjustments in bodily function and appearance [3], which affect physical, psychological, and social aspects of life [4–8], as well as quality of life [9, 10]. Challenges related to adaptation may include managing leakage, gas, and odour, practical stoma care, body image, sexual difficulties and activities involving social interaction. Despite these challenges in adaptation, few intervention studies have focused on these adjustment-related issues [5, 11].

Health authorities advocate for the use of patient-reported outcome data, defined as “reports coming directly from patients about how they function or feel in relation to a health condition or intervention, without interpretation by anyone else” [12]. Patient-reported outcome measures (PROMs) systematically track patients’ progress, often utilised by a clinical feedback system (CFS). The routine collection and use of PROMs aim to enhance patient-centred care and inform value-based initiatives [13–15]. Although their use has increased, the extent of implementation varies.

The use of PROMs has been studied across various conditions and clinical settings. Generally, communication between patients and clinicians has improved [16–19], including an enhanced focus on consultations [20]. In both somatic and psychiatric healthcare settings, PROMs may also facilitate self-reflection, health awareness, self-efficacy, and active patient involvement [17, 19–21]. Findings regarding collaboration between patients and healthcare professionals have been mixed [20, 21]. In a systematic review of qualitative studies among various somatic care settings, Campbell et al. [20] noted that patients may have unrealistic expectations of care and that using PROMs may not be suitable for all patients. Rognstad et al. [22] identified a small positive effect on treatment outcomes for common mental health disorders. Additionally, PROMs have demonstrated a positive effect on quality of life among various conditions and settings [16] and in cancer care [18].

A literature review by Kittscha et al. [11] identified a lack of intervention studies addressing adjustment problems in ostomy care. In Norway, SCNs play a central role in the routine follow-up of patients after ostomy surgery, which is part of the specialist health care service. The Ostomy Adjustment System, which was developed as the first clinical feedback system (CFS) in ostomy care, was designed to collect patient-rated data for SCNs to review in routine care. This CFS allows patients to prepare for follow-up consultations with SCNs and effectively communicate their experiences and challenges to their SCNs, aiming better to address patients’ needs during the adjustment process [23]. A subsequent quantitative study of patients’ experiences with CFS reported that patients were satisfied with their follow-up care, received personalised information, were involved in treatment decisions, and benefited from consultations [24]. However, there is still a gap in the research literature concerning what makes CFS helpful.

This article is part of a larger study aiming to explore the use of CFS from the patients’ perspective in-depth. Patients’ experiences using the CFS in prior consultations indicate that engaging with the CFS had personal utility across many dimensions, varying in strength and significance for each individual. Although not everyone grasped the purpose, it was part of a preparatory learning process for the consultation and the adjustment process. It triggered reflection and self-awareness and served as a means of communication, with the potential for further follow-up [25].

In this article, we aimed to address the following research question: How do patients experience using CFS during follow-up consultations in ostomy care?

## Methods and materials

### Context

In Norway, SCNs play a central role in the routine follow-up consultations of patients after ostomy surgery, which is part of the specialist healthcare service. SCNs follow national professional recommendations for follow-up consultations, which are scheduled at 3 weeks, 3, 6, and 12 months postoperatively and then annually. They also provide a low-threshold service. These follow-up consultations include (1) clinical control of the ostomy, skin, and ostomy equipment and (2) discussions with the patient, including health education and guidance [26]. From 3 months onwards, the CFS is applied as part of all routine follow-up consultations, and the questionnaire is sent out 14 days in advance. A total of 14 SCNs were employed at the four outpatient clinics at the time the study was conducted.

**Fig. 1:**
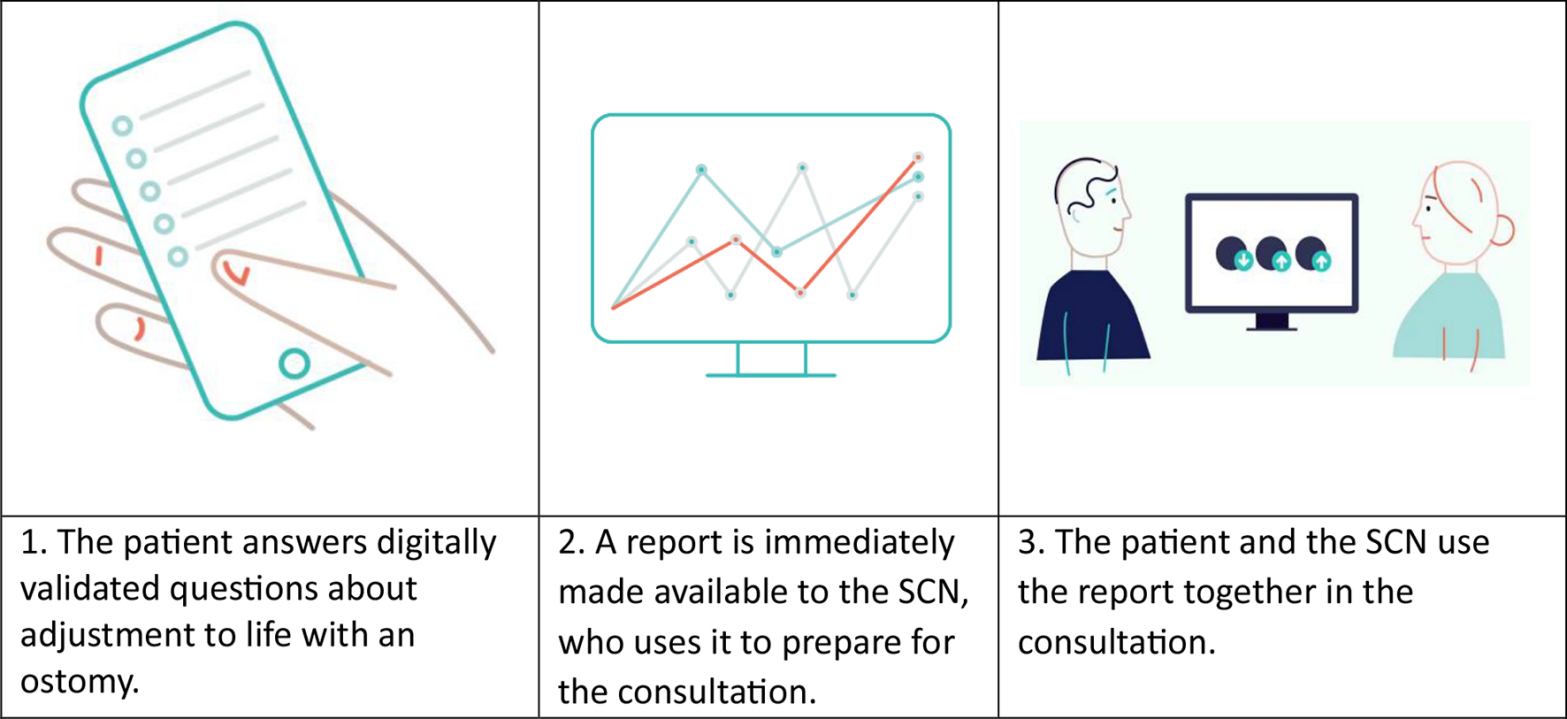
Use of the ostomy adjustment system

The Førde Hospital Trust developed the Ostomy Adjustment System to improve consultation, patient involvement, and adaptation to life with an ostomy. Items were generated based on the needs of patients and SCNs [10, 23, 27], standardised for the Norwegian population [28], and tested in clinical development studies [24]. The CFS contains a sociodemographic and clinical form: the Ostomy Adjustment Scale, a six-point Likert-type scale reflecting the adjustment process [29], and the Coop-Wonca Chart, a five-point Likert scale for HRQoL [30]. The CFS was first implemented at an outpatient clinic in 2017. Version 2, which consists of up to 86 items distributed across multiple scales [27], was rolled out to three additional hospitals within the same health authority in 2022. Before each consultation, starting from 3 months after surgery, patients complete the CFS electronically via smartphone, PC, or tablet [23] (see Fig. 1).

The CFS generates visual reports with graphs and bars summarising the patient’s adjustment and development throughout follow-ups. SCNs can access the complete list of patients’ responses to each item. Immediately after the patient completes the questionnaire, the reports become available to the SCNs, but not the patients, and can be viewed by both during consultations.

Study design.

An exploratory, inductive qualitative design was used to explore participants’ experiences with the CFS during consultations, thereby addressing the second research question (RQ) within a larger study. The first RQ, concerning the use of the CFS in preparation for those consultations, has been addressed elsewhere [25].

### Participants and recruitment

​Patients were recruited from four outpatient clinics across four hospitals. A SCN in a key coordinating role oversaw the recruitment process and maintained participant anonymity in collaboration with a locally responsible SCN at each hospital. This approach ensured that each SCN distributed participation invitations to achieve diversity in gender, age, type of ostomy, number of consultations, and scores on the Ostomy Adjustment Scale while also ensuring adequate participation from each hospital in the overall sample. The SCNs at each hospital sent invitations to patients, who responded directly to the first author. Inclusion criteria for the study were as follows: 18 years or older; with a colostomy, ileostomy, or urostomy; having had the ostomy for more than 3 months; having completed outpatient follow-up at 3 months; and being able to speak and write Norwegian.

Of 30 invited patients, 27 responded (14 female and 13 men), hereafter referred to as participants. The average age was 59 years (range 23–83). Most had curative treatments; a few were receiving palliative care. Time since surgery ranged from 3 months to 12 years. All participants had attended at least two follow-up consultations at the time of the interview. The interviews were conducted at varying intervals following the most recent consultation. Patient participants characteristics are provided in Table 1.

**Table 1:** Demographic characteristics of the patient participants

Patient No.	Sex	Age	OAS score^1^	Time since surgery in months^2^	Number of consultations using OAS	Ostomy type	Hospital	Diagnosis
P1	f	40s	≤ 2.9	18	2	Colostomy	1	Bacterial
P2	f	40s	≤ 2.9	12 years	5	Urinary reservoir/Colostomy	1	Functional diarrhoea
P3	m	70s	≤ 2.9	10	3	Colostomy	1	Incontinence
P4	m	70s	≤ 2.9	6 years	6	Urostomy/Colostomy	1	Radiation damage
P5	f	80s	≤ 2.9	28	6	Colostomy	1	Ca. recti
P6	m	70s	≤ 2.9	14	2	Colostomy	2	Ca. recti
P7	m	60s	≤ 2.9	13	3	Colostomy	1	Ca. recti
P8	m	70s	≤ 2.9	13	3	Urostomy	2	Ca vesica
P9	f	70s	≤ 2.9	15	3	Colostomy	2	Ca. recti
P10	m	60s	≤ 2.9	10	2	Urostomy	4	Ca. vesica
P11	f	70s	≤ 2.9	9	2	Colostomy	4	Diverticulitis
P12	m	40s	≤ 2.9	15	3	Ileostomy	2	IBD
P13	f	30s	≥ 3.0	10 years	6	Ileostomy	1	Hirschsprung disease
P14	f	40s	≤ 2.9	19	2	Ileostomy	1	IBD
P15	m	50s	≥ 3.0	14	2	Ileostomy	3	Ca. recti
P16	m	70s	≤ 2.9	14	2	Urostomy	3	Ca. vesica
P17	m	50s	≤ 2.9	27	4	Urostomy	2	Ca. vesica
P18	f	80s	≤ 2.9	14	3	Colostomy	2	Ca. recti
P19	f	60s	≤ 2.9	17	2	Urostomy	4	Bladder exstrophy
P20	f	60s	≥ 3.0	3	3	Colostomy	3	Diverticulitis
P21	f	20s	≤ 2.9	34	5	Ileostomy	1	IBD
P22	f	30s	≤ 2.9	18	3	Ileostomy	1	IBD
P23	f	60s	≤ 2.9	11	2	Colostomy	4	Incontinence
P24	m	50s	≥ 3.0	13	2	Colostomy	3	Ca. recti
P25	f	60s	≥ 3.0	15	3	Colostomy	3	Diverticulitis
P26	m	60s	≤ 2.9	4	2	Colostomy	4	Ca. recti
P27	m	30s	≥ 3.0	12	2	Ileostomy	4	IBD

^1^ Mean score ranging from 1 to 6. There is variation in the dataset. Scores lower than 4.35 indicate good adjustment, 2.67 to 4.34 some challenges, and 2.66 to 1 high adjustment

^2^ Some patients had received an ostomy before the use of CFS in stoma care from 2017

### Data collection

The interview guide was developed based on theoretical considerations for conducting exploratory interviews, prior work and a literature review. It was refined for content, clarity, and fluency through expert discussions within the research team and the affiliated research group linked to the strategic research program. Two pilot interviews led to further refinement. The guide covers two domains: digital questionnaire and follow-up, focusing on communication, relationship, interaction, participation, and person-centredness (see Supplementary file 1). LAJ conducted individual semi-structured interviews in a private room at a public institution or the participants’ homes between September 2023 and February 2024. Minor changes were made after a few interviews, followed by another pilot interview. All interviews were recorded, averaging 72 min (ranging from 51 to 107 min). Three participants had supplementary telephone interviews.

### Data analysis

The data were analysed using Braun and Clarke’s reflexive approach to thematic analysis (RTA). This six-phase process includes familiarisation with the dataset, systematic coding, and developing, reviewing, and refining themes before producing the final report. In this approach, we as researchers view coding as an organic and flexible process requiring detailed engagement with the data; our subjectivity is integral to the analysis, and we reflect critically on our roles, practices, and processes [31]. LAJ conducted the analysis closely with AMS, involving patient user representatives and the research team at various stages to seek new insights and validation. The entire research team convened in person on multiple occasions during the analysis process to review initial and revised themes and to provide feedback. LAJ held meetings with patient user representatives to discuss the thematic abstraction and the refined themes and to evaluate illustrative quotations. The entire dataset was analysed guided by two RQs described under the study design. Details on the analysis process can be found in Supplementary File 2.

### Research team

LAJ, a Ph.D. candidate, is a trained therapist and psychiatric nurse with experience in qualitative research. AMS is a Professor of Nursing Science with extensive experience in qualitative research. KLI, who holds a Ph.D. in stoma care nursing, works as an SCN, and JRA is also a Professor of Nursing Science. All authors have critically reflected on their preconceptions and interests, approaching the data with an open mind.

Three patient user representatives, recruited by Førde Hospital Trust, participated in pilot interviews and were involved in the analysis process.

### Ethics

All procedures were conducted in accordance with the 1964 Helsinki Declaration and its later amendments. This study was approved by the regional ethics committee (ID 593949) and the local data protection officers (references 4183). Anonymised transcripts were stored on secure servers. All participants received written information about the study’s purpose, anonymity, confidentiality, data security, and their right to withdraw at any time without consequences. Written informed consent was obtained from all participants prior to the interviews. The interviewer asked participants about their interview experiences, and all reported positive experiences. No distress was noted.

## Results

The overarching theme “A flexible, engaged, person-centred follow-up”, along with four themes, was developed through analysis: (1) Explicit and implicit use of information in consultations, (2) A springboard for deeper dialogue on sensitive issues, (3) Reassurance that changes in health status will be captured and adequately assessed, and (4) Utility depends on continuity of use.

Participants experienced receiving care tailored to their needs and felt they were at the centre of the follow-up sessions. There were variations in how patients experienced the responses being utilised, articulated, and visualised by the SCN during consultations. Participants reported that their responses to the CFS were referenced implicitly and explicitly, and they expressed a preference for direct communication. They found personal value in using the CFS, which facilitated communication, particularly on sensitive topics. The SCN’s use of responses and expertise provided a sense of security when assessing health status. Regular use of the CFS and a clear understanding of its purpose enhanced its utility and enabled participants to participate actively in their treatment. Although participants varied in how strong or significant they found each theme, all themes were represented in all interviews. The degree of utility of the CFS depended on the participant’s needs and preferences in their adjustment to living with a stoma.

**Fig. 2:**
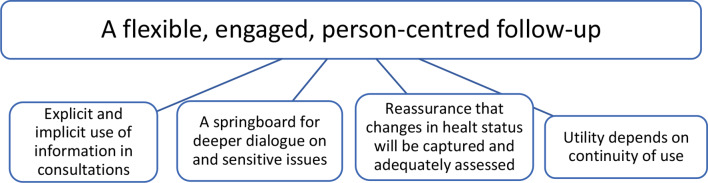
Overview of thematic structure of findings: The overarching theme along with four themes

### Explicit and implicit use of information in consultations

Participants varied regarding how they experienced their responses being used, articulated, and displayed by the SCN during consultations. The SCN could refer to patients’ information as a comprehensive picture; however, the participants experienced that the SCN explicitly referenced the information by directly asking about specific items or scores, typically as part of the initial conversation in the consultations. The participants observed that the SCN focused on prominent issues. Several participants noted that the SCN displayed the responses on a screen in graphs or bar charts during the consultations. The visualisation helped them reinforce their awareness of their situation and why and how the CFS was used. One participant described the conversation as follows:

It feels like a real conversation. It’s not merely about lighting up red [referring to low adjustment scores]. It’s more about identifying the problems, finding solutions together, and improving my situation in the long term. That’s how I feel when she [the SCN] uses my responses in the consultations (P1; female, 40s)

Participants also experienced that the SCN used the information implicitly during consultations without mentioning details, such as items or scores, or displaying the report on the screen. In the dialogue, the SCN could subtly steer the conversation towards essential topics; these participants interpreted the information they had provided as indirectly supporting these discussions. For some participants, it was unclear how much their responses were used in the consultation. One participant reflected:

Now that we’ve discussed it, maybe she actually did base her questions on what I had answered. At the time, I didn’t think about it at all, but now, reflecting on it this way, that seems likely. We discussed what was necessary and what I needed to talk about (P18; female 80s)

Some other participants perceived that the SCN did not use the information provided as a foundation for the conversations. Instead, they thought the SCN asked questions spontaneously and based on their professional knowledge. The participants preferred clear and direct language for all topics over vague and unclear approaches from the SCN.

### A springboard for deeper dialogue on sensitive issues

The CFS’s structured responses facilitated conversations by serving as a springboard for deeper dialogue on important issues and as prompts for further communication. Many participants found the CFS to be an effective tool for communication, particularly for discussing sensitive topics such as sexuality, appearance, and self-esteem. It enabled them to articulate how they related to their ostomy and their emotional responses to living with it. In many cases, SCNs’ focus on prominent issues led to a discussion of topics the participants themselves wanted addressed during the consultations.

Many participants found that, especially when sensitive topics were being addressed, communicating through a structured questionnaire was more accessible than an entirely open-ended discussion. They appreciated the questionnaire as an opportunity to express feelings they might otherwise have hesitated to voice. They found it natural and comfortable that a screen was used to display the responses. Alternating their gaze between the screen and the SCN reduced the awkwardness of direct eye contact during difficult conversations about intimate matters. This visual aid subtly guided the conversation between the participant and the SCN, helping them navigate sensitive topics and especially helping participants engage in challenging discussions. One participant shared,

I don’t always feel brave enough to say things face-to-face, but after I’ve filled out the form, the conversation starts naturally. It’s easier to keep my eyes on the screen rather than make direct eye contact. It helps me start talking about things that are a bit difficult or private… like feeling unattractive (P13; female, 30s).

The screen’s presence in the room created a sense of comfort, making difficult conversations more approachable. There was a consensus among participants that the SCN should take the lead in initiating discussions on particularly sensitive topics, such as sexual health and mental well-being. They expressed a desire for the SCN to proactively address these issues, even if they were not explicitly highlighted in the questionnaire. A more direct approach could have made it easier for some to open up.

I was expecting a more straightforward approach. I would have opened up. I am prepared to discuss it since I have already answered questions on the topic. After all, I still have the opportunity to respond. I have a voice and a choice (P17; male 40s)

Using the CFS was instrumental in focusing consultations on what was most important to the participants. Their investment of time in completing the CFS allowed them to get the most out of the consultation time allotted.

The value of sufficient consultation time was a recurring theme among the participants. They deeply valued access to specialist expertise, which they considered crucial for addressing the technical aspects of their care and the broader challenges of living with an ostomy. This access to expertise was vital for learning and receiving guidance on various aspects of life affected by their condition.

That time [the consultation] becomes so important for me to have access to that expertise. There are many aspects to it, the technical, the equipment, and all that, but then there’s everything else… life. All I need to learn and get guidance on (P14; female, 40s).

One participant even mentioned that the consultations felt more effective because there was less *irrelevant talk* (P12), indicating that the structured approach helped eliminate unnecessary small talk and kept the focus on what truly mattered.

### Reassurance that changes in health status will be captured and adequately assessed

Participants experienced that using the CFS allowed them to monitor their adjustment to life with an ostomy. Combined with the expertise of the SCN, the use of the CFS was crucial in detecting changes that required attention, whether these were changes the participants had overlooked, were unaware of, or lacked the knowledge to evaluate. Many valued this combination as a safeguard against potential adverse developments in their physical and mental health. Having the SCN monitor their health status and adaptation process through the CFS provided a strong sense of security. Participants appreciated knowing that any changes or problem areas could be quickly identified and addressed. This confidence in preventing adverse outcomes gave them peace of mind.

When I know that the nurse is going through my responses, I feel much more secure that anything needing attention will be picked up. She has the expertise to recognise it… using the questionnaire gives me such a strong sense of security (P22; female 30s)

Many participants noted that using the CFS during consultations increased their awareness of and knowledge about changes, including positive ones. Seeing these improvements visually represented on the screen motivated them and provided hope throughout the adaptation process, fostering a sense of control. Initially, many areas were marked as needing attention, but over time, as participants reviewed the reports, they noticed improvements that they themselves had not spontaneously detected. This reinforced their belief that things were improving and gave them a sense of accomplishment.

In the beginning, everything was red [indicative of low adjustment]. Even though I didn’t feel that things had changed, I saw that things had improved. It was actually getting better. That helped a bit… a kind of mastery (P13; female, 30s)

Another participant echoed this, mentioning that comparing scores over time became a source of motivation. Seeing tangible evidence of positive changes encouraged them to continue adapting to life with an ostomy:

The scores were compared. That was a motivation in itself then… to see that things had actually changed for the better (P15; male 50s)

Participants also recognised that their needs could change over time. Integrating the CFS into consultations enabled them to address or revisit issues that might have emerged later or had not been previously discussed. Immediately after surgery, many participants focused on addressing physical health concerns and finding functional equipment. At the same time, specific challenges such as sexual difficulties or body acceptance in social situations were not prioritised. The need to discuss more personal and emotional challenges often arose later, particularly during annual consultations.

The CFS and SCN partnership offered participants a sense of security and control in their adaptation journey by providing a structured way to monitor changes and a safe environment for discussion.

### Utility depends on continuity of use

Participants who understood the purpose of the CFS and experienced consistency and continuity in its use during routine follow-ups found significant personal utility in using the CFS. This utility was achieved through reflection and increased self-awareness in the course of preparation for consultations and consequently through more active participation during consultations themselves. One participant reflected:

I have to prepare myself and be more active. Then, I became more active in the consultation. I engage more, in a way, in myself. Yes, in the right way (P11; male, 50s).

The understanding of purpose, the routine of completing the questionnaire before each consultation, the SCN’s active use of the information in dialogue, and the displaying of the reports during each session collectively facilitated mutual communication and gave participants a sense of security. An active patient role promoted and strengthened shared decision-making in consultations.

I feel I get to decide… yes, together with the SCN. I am essentially enabled to make decisions based on my own experience, using the CFS and the information I receive. […] When I answer the questions and we discuss them in the consultation, I get the information I need. Then, it becomes easier for me to decide or make choices, so to speak. […] When we schedule a new appointment, it’s something we come up with together, but I’m the one who says what I need (P7; male, 60s)

The experience of regular use varied in intensity among participants from different hospitals. A few participants who did not adopt an active patient role experienced a lesser sense of utility in using the CFS. These patients struggled with orientation, understanding health information, and managing their ostomy, and their scores on the adjustment scale remained low after baseline.

The participants’ active patient role was associated with use and perceived utility, promoting and strengthening shared decision-making in consultations.

## Discussion

This study is the first to examine how ostomy patients experience using CFS as part of routine care. Participants experienced follow-up consultations as flexible and sensed that SCNs engaged with them as individuals, not just as cases to be managed. However, there were variations in how participants experienced the SCNs’ use of their responses. They experienced personal utility in using the CFS, and regular use - combined with understanding the purpose - enhanced this utility and enabled them to participate actively in their treatment.

### CFS benefits for patients

The use of CFS in consultations appears to make a valuable contribution by facilitating communication, particularly on sensitive topics such as sexuality, self-esteem, and body image. It helps to explore problem areas, and the SCN’s use of responses and expertise provides a sense of reassurance when the participant’s health is assessed. However, there were variations in how they experienced the responses being used, articulated, and visualised by the SCNs.

This study highlights nuances of communication and focus during consultations, reflecting findings from previous studies from various clinical settings [20, 21]. In line with previous findings, our results show that using CFS is not a neutral act or an objective process [15, 25], and there is subjectivity in how participants find the tool valuable - just as there appears to be subjectivity in how SCNs apply the use in clinical practice. Interestingly, some participants felt their responses were not utilised and perceived that SCNs relied more heavily on professional expertise and judgment to determine what the participants needed during the consultation. These are noteworthy findings and warrant further exploration. Notably, these participants came from hospitals where the CFS had been implemented most recently and where staff numbers were low, possibly pointing to implementation issues [32].

Defining one way to use a CFS with all patients will probably not lead to beneficial patient experiences, as patients require different approaches based on their capacities. However, these findings can offer some general implications and suggestions on how to use a CFS in a manner that is helpful to patients. Regardless of how and to what degree CFS was used, all patients wanted the information to help them adapt to life with an ostomy. They wanted problem areas to be discussed, whether sensitive or not and preferred direct use of the responses and open communication about problem areas, including sensitive topics. This finding underscores the importance of communication skills for SCNs [33] and other advanced competencies [34].

Participants desire SCNs to initiate the dialogue when they find it difficult, as demonstrated in a study highlighting the challenges of discussing overweight– a sensitive topic– with GPs [35]. Findings from patients’ experiences of answering the CFS prior to consultations indicated that specific questions, particularly those concerning sexuality, were challenging to answer in a way that provided a comprehensive picture of their situation [25]. These findings suggest that SCNs should not draw firm conclusions based solely on the CFS responses. Instead, they should take the initiative to explore these topics further during the consultations, with an understanding that patients find it difficult to articulate the challenges they face [36].

### Potentially enhancing utility and person-centredness

The findings suggest that continuity and consistency in using CFS during follow-up and understanding its purpose positively impacted participants’ perceived personal utility and enabled them to take an active role in their treatment. A study that used the CFS in a mental health outpatient clinic found that a minimum requirement for successful implementation is that patients understand the purpose of the system and that their responses are acknowledged and recognised [37]. Furthermore, the authors emphasise that therapist-patient dyads should explore and negotiate how and when to use the CFS [37] - an approach that may also be relevant in the patient–SCN collaboration within ostomy care. Regardless, consistent use of the system throughout the follow-up period is a fundamental prerequisite.

Beyond its potential to enhance patients’ perceived personal utility, findings from pre- and during-consultation experiences indicate that patients show a fundamental willingness and positive attitude toward the CFS [25]. This, in turn, suggests that for SCNs, using the CFS in collaboration with patients who hold such an attitude may strengthen their ability to provide person-centred care, provided the system is used purposefully and consistently [25, 38]. At its core, this person-centred practice has a therapeutic intent translated through relationships built on effective interpersonal processes, within which the CFS may also be regarded as a supportive tool [38].

Monitoring the adjustment process to improve it may address patients’ needs. However, using the system without further exploration, dialogue, and collaboration may lead to experiences of it being a meaningless bureaucratic exercise and raise ethical dilemmas.

The findings suggest there is no way to use CFS that fits all patients, as they have diverse needs and capacities. Nevertheless, the results provide confirmatory evidence that using the CFS in ostomy care can increase the likelihood that SCNs tailor care to patients’ subjective needs and support the provision of person-centred care [13, 19].

### Limitations

The rich and varied data illuminate multiple aspects of the RQ. To enhance reliability, we meticulously outlined the process of interpretation, allowing readers to trace the analytical pathway [31]. However, retrospectively exploring experiences can be challenging, as it may not capture immediate experiences accurately. In line with the nature of qualitative research, the results cannot be generalised to all users of the CFS and must be understood in context. Furthermore, participants were recruited with instructions to ensure variation, not randomly, which introduces potential reservations regarding who was chosen and why. This limitation does not undermine the significance of our results, which inform and expand upon the existing literature.

### Relevance to clinical practice and further research

Defining one way to use CFS with all patients may not lead to beneficial patient experiences as patients have varying capacities and needs. However, the findings in this study can offer some general implications and suggestions on how to use CFS in a manner that is helpful to patients.

PROMs are good starting points for discussing what is important. They must be explored further in consultations, preferably by SCNs explicitly referring to the answers and supporting them with visual representations on the screen. Patients prefer direct communication, and SCNs must take the lead in discussing sensitive topics. If a CFS is used regularly in clinical practice, there may be greater potential for more patients.

It is important to explore why some patients do not find the CFS useful and how this may relate to their level of health literacy. Further research should investigate SCNs’ attitudes and perspectives on using the CFS in ostomy care. Longitudinal studies should be conducted to gain deeper insights into the role of CFS in the treatment process.

## Conclusions

The findings indicate that using the CFS holds personal value for patients and appears promising in ostomy care. To be helpful to patients, the CFS should be used in a manner that is meaningful to patients and attuned to individual needs, preferences, and the personal adjustment process. It can strengthen SCNs’ ability to tailor care to patients’ needs and support person-centred care. However, the study also highlights the need for greater emphasis on regular use in clinical practice to reach its full potential.

## Electronic supplementary material

Below is the link to the electronic supplementary material.

Supplementary Material 1

Supplementary Material 2

## Data Availability

The dataset supporting this study’s findings is not openly available due to reasons of sensitivity but is available from the corresponding author upon reasonable request.

## Abbreviations

SCN: Stoma care nurse
CFS: Clinical feedback system
OAS: Ostomy Adjustment System.
PROM: Patient-reported outcome measure
